# Progressive multifocal leukoencephalopathy in a patient with multiple myeloma: a case report and analysis of the FDA adverse event reporting system

**DOI:** 10.3389/fneur.2023.1098930

**Published:** 2023-05-04

**Authors:** Elise Jonasson, Ronald Antulov, Per Trøllund Pedersen, Tobias Sejbæk

**Affiliations:** ^1^Department of Hematology, Hospital South West Jutland, University Hospital of Southern Denmark, Esbjerg, Denmark; ^2^Department of Radiology and Nuclear Medicine, Hospital South West Jutland, University Hospital of Southern Denmark, Esbjerg, Denmark; ^3^Department of Regional Health Research, University of Southern Denmark, Esbjerg, Denmark; ^4^Department of Neurology, Hospital South West Jutland, University Hospital of Southern Denmark, Esbjerg, Denmark

**Keywords:** progressive multifocal leukoencephalopathy, multiple myeloma, PML, case report, immunosuppression, FAERS

## Abstract

This paper demonstrates a case of progressive multifocal leukoencephalopathy (PML) in a patient with multiple myeloma (MM) treated with nine different MM therapies. This case report contributes to the already published 16 cases of PML in patients with MM. Additionally, this paper presents an analysis of cases from the United States Food and Drug Administration Adverse Event Report System database (n = 117) with a description of demographics and MM-specific therapies. Patients with MM, that developed PML, were treated with immunomodulatory drugs (97%), alkylating agents (52%), and/or proteasome inhibitors (49%). Prior to PML diagnosis, 72% of patients received two or more MM therapies. These results indicate that PML in MM is underreported and could be related to treatment with multiple immunosuppressive therapies rather than MM as a disease itself. Physicians should be aware of potential PML in the late stage of heavily treated MM patients.

## Introduction

Multiple myeloma (MM) is the second most common hematological malignancy, primarily occurring in elderly and middle-aged people with ~85% diagnosed after the age of 55 years. Over the past three decades, MM treatment has improved leading to increased survival, first with high-dose chemotherapy (HDT) complemented by autologous stem cell transplantation (ASCT) and later with improved treatment regimens with immunomodulatory drugs (IMiD) including thalidomide, lenalidomide, and pomalidomide; proteasome inhibitors (PI) including bortezomib and carfilzomib; and monoclonal antibodies including daratumumab and elotuzumab (1–3).

Multiple myeloma is an incurable disease but sensitive to many different types of treatment. Early in the disease course, patients can experience longer periods of remission, but later on, the time between relapses will be shorter and patients will often need continuous treatment in order to control the malignant plasma cell clone. In recent years, most new treatment regimens have included continuous treatment and maintenance treatment with novel antimyeloma drugs compared to fixed-duration approaches (3–5). Maintenance with lenalidomide after ASCT leads to prolonged progression-free survival and, in some studies, prolonged overall survival (6–9). In addition, studies have demonstrated that daratumumab used in combination with lenalidomide or bortezomib given until progression is associated with a lower risk of disease progression and death (10, 11). This variety of new drugs and continuous treatment regimens lead to longer survival among MM patients but also to a prolonged state of severe immunosuppression.

Progressive multifocal leukoencephalopathy (PML) is an opportunistic central nervous system (CNS) infection caused by John Cunningham (JC) virus. Primary infection with the JC virus is common in the general population and likely occurs in the stromal or immune cells of the upper respiratory system. The virus is later trafficked by lymphocytes into the bone marrow or kidneys where it persists in a latent stage. Seroconversion increases with age and reaches ~60–80% at the age of 70 years. The pathogenesis of PML is the reactivation of the JC virus in glial cells in the CNS causing multifocal destructive brain lesions in patients with severe immunosuppression (12, 13). PML in patients with MM is rare but still a severe disease leading to disability and death. Currently, only 16 published cases, written in English, are available (13). This manuscript presents an additional case and a unique analysis of reported cases from the United States Food and Drug Administration (FDA) adverse events reporting system (FAERS) database.

## Case presentation

**Figure 1:**
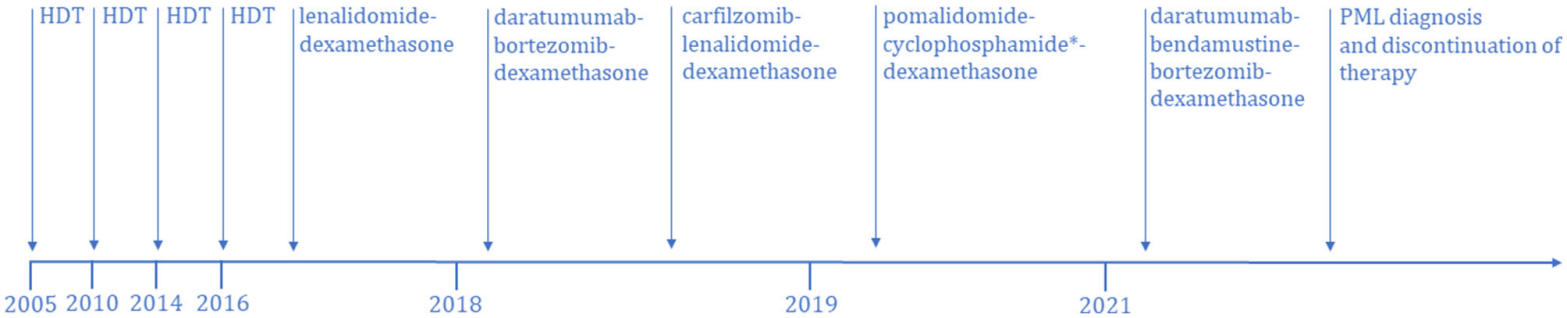
Treatment timeline of multiple myeloma-specific therapies from disease onset until the diagnosis of PML. HDT, high dose therapy. *Nine series without cyclophosphamide.

A 39-year-old woman was diagnosed with IgG-kappa MM in 2005. The patient received HDT with ASCT four times over a period of 11 years. First ASCT was at the time of diagnosis and the second ASCT was 5 years later in 2010 due to MM relapse. Prior to the first and second ASCT, the patient was treated with cyclophosphamide and dexamethasone followed by high-dose melphalan. The third ASCT was 4 years later in 2014, and prior to this, the patient was treated with cyclophosphamide, bortezomib, and dexamethasone followed by high-dose melphalan. Treatment before the fourth ASCT, in 2016, was bortezomib, thalidomide, and dexamethasone followed by high-dose melphalan (Figure 1).

After the fourth ASCT, the patient was treated with 20 series of lenalidomide and dexamethasone to maintain remission. Due to disease progression in early 2018, treatment was switched to daratumumab, bortezomib, and dexamethasone, but after only one treatment, there was disease progression. Treatment was then changed to carfilzomib, lenalidomide, and dexamethasone, and eight series were given. The patient developed carfilzomib toxicity, and the treatment was halted at the end of 2018. Treatment was changed to pomalidomide, cyclophosphamide, and dexamethasone, and 21 series were given from 2019 to 2021. Eight of the 21 series were without cyclophosphamide due to severe neutropenia.

**Figure 2:**
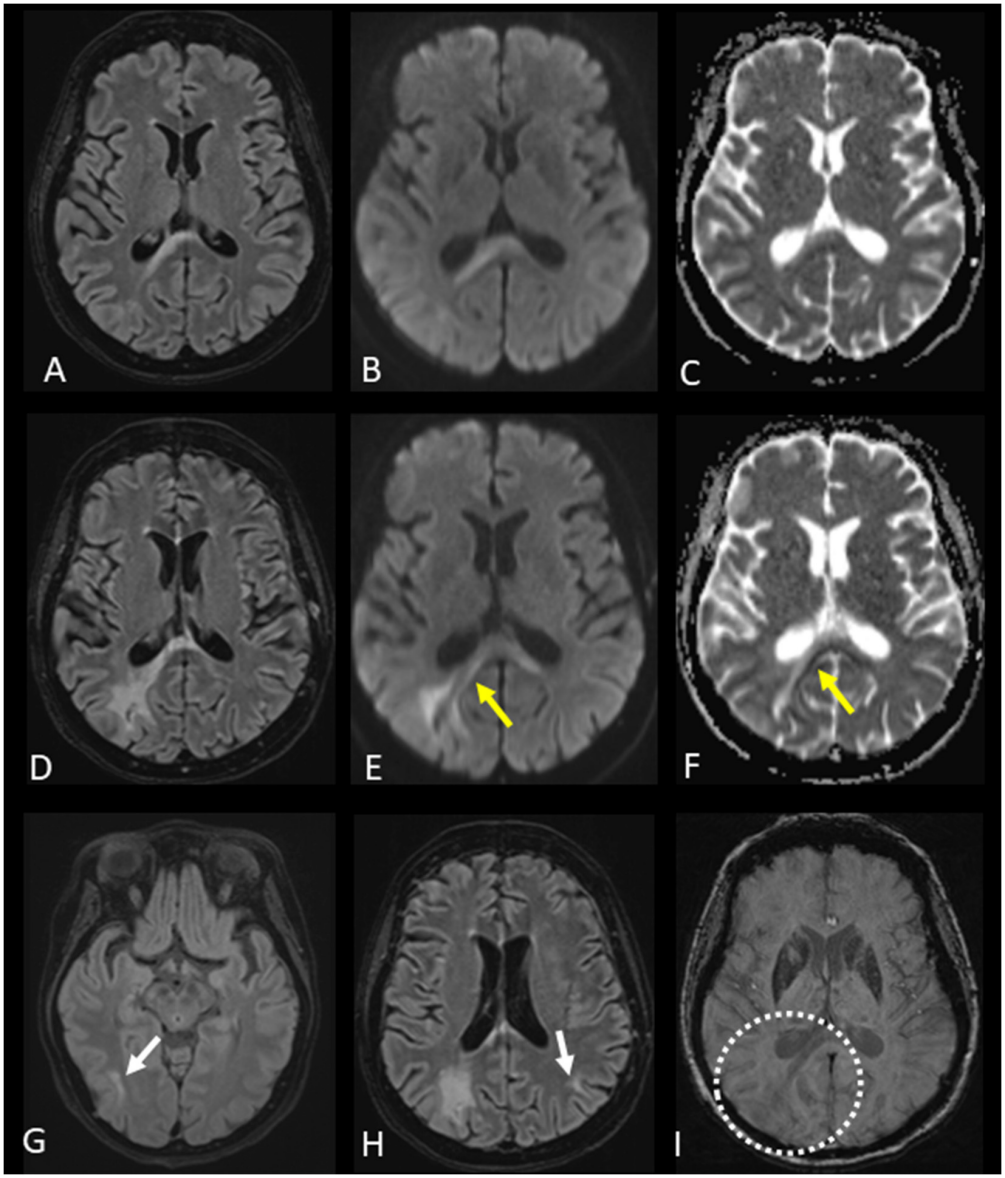
Evolution of progressive multifocal leukoencephalopathy (PML) and magnetic resonance imaging (MRI) changes. **(A–C)** First brain MRI with fluid-attenuated inversion recovery (FLAIR), diffusion-weighted imaging (DWI) b 1,000 images, and the apparent diffusion coefficient (ADC) map presented from left to right columns showing a lesion of the splenium of the corpus callosum, predominantly on the right side of the increased signal on FLAIR and DWI b 1,000 images, without restricted diffusion on the ADC map. **(D–F)** Second brain MRI with FLAIR and DWI b 1,000 images, as well as the ADC map, presented from left to right columns indicating an increase in the size of the corpus callosum lesion along with partial peripheral restricted diffusion visible on the DWI b 1,000 images and ADC map (yellow arrows). The newly appearing peripheral changes on diffusion images were indicative of PML. **(G, H)** FLAIR images of the second brain MRI demonstrating new lesions in the right temporal lobe and left parietal lobe (white arrows). **(I)** Susceptibility-weighted image of the second brain MRI without cortical-subcortical junction band of low signal intensity related to the expanding lesion from the corpus callosum (dotted circle). These susceptibility brain changes, which most likely represent iron accumulation, were described only in a part of PML patients.

In 2019, 2 years prior to PML diagnosis, the patient had intracranial myeloma which regressed on treatment. In 2021, due to urinary retention, a history of intracranial disease and cognitive worsening a magnetic resonance imaging (MRI) examination of the brain and lumbar puncture were performed. Cerebrospinal fluid (CSF) was with normal levels of leukocytes ( ≤ 5^*^10^6^/L), and CSF flow cytometry revealed no plasma cells. CSF was not analyzed for the JC virus. Brain MRI showed no intracranial myeloma-related findings, but a non-specific lesion involving the splenium of the corpus callosum that could possibly represent a subacute ischemic change, a cytotoxic lesion of the corpus callosum, or a low-grade glioma was described (Figures 2A–C). The patient was afterwards treated with daratumumab, bendamustine, bortezomib, and dexamethasone for two series but stopped due to worsened general condition during the summer of 2021. The patient deteriorated cognitively and was bedbound most of the day. The symptoms debuted 1 month prior to the first MRI and progressed until death 5 months later. On neurological examination, the patient had left-sided hemianopsia.

This led to a new brain MRI, performed 12 weeks after the first brain MRI, showing progression of the corpus callosum lesion along with new brain lesions (Figures 2D–I). The appearance of new brain lesions, along with the enlargement of the corpus callosum lesion and related changes on diffusion-weighted images, were highly suggestive of PML. A lumbar puncture was performed, and 51,900 copies/ml of JC virus were found in the CSF, confirming the diagnosis of PML.

Due to the PML diagnosis and the rapidly deteriorating general condition of the patient, no further MM treatment was given. Progressive cognitive impairment followed, and the patient died four weeks after the PML diagnosis.

## FAERS database

**Figure 3:**
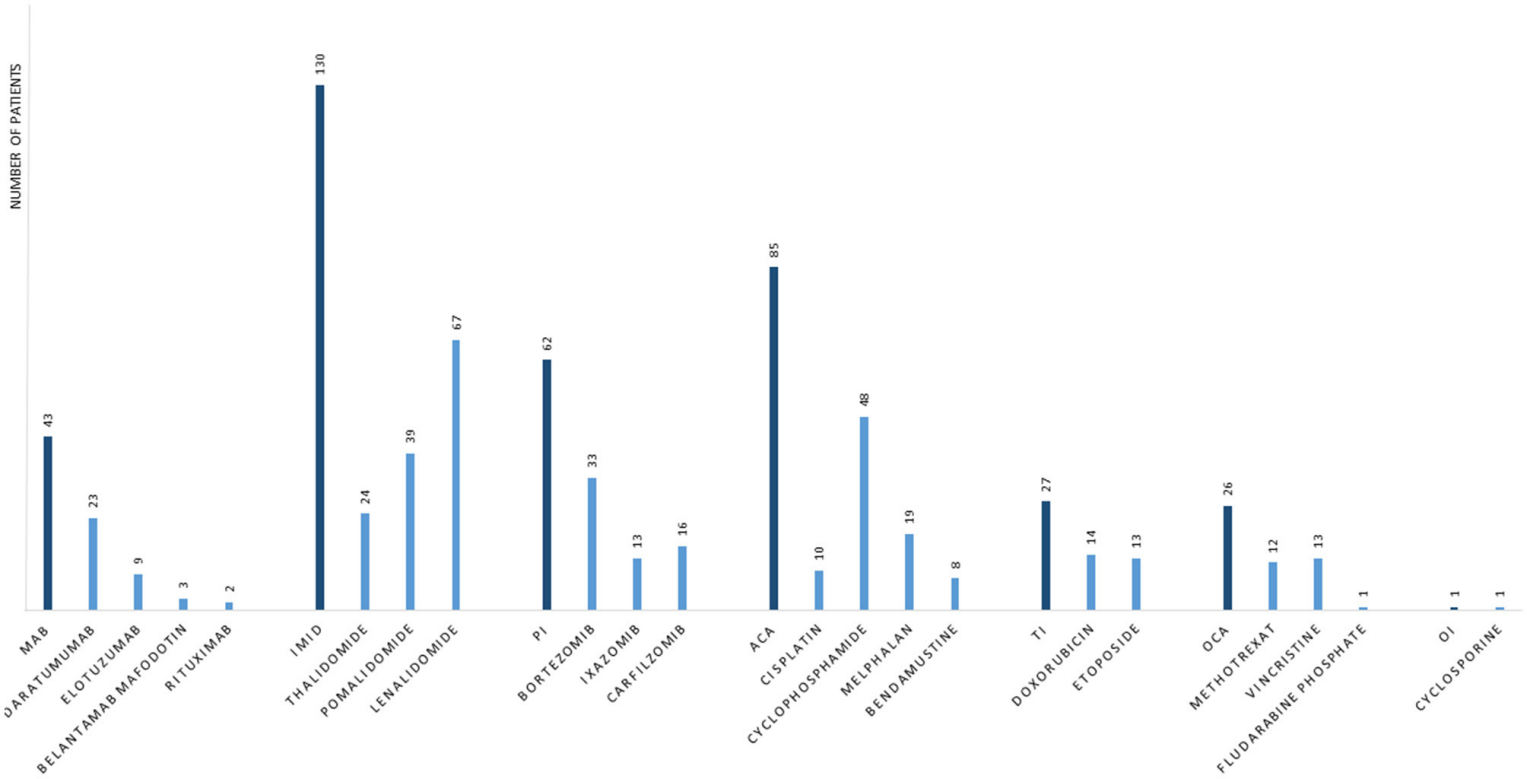
Frequency of specific multiple myeloma therapies in cases that developed progressive multifocal leukoencephalopathy. Data are from the United States Food and Drug Administration adverse events reporting system database. MAB, monoclonal antibodies; IMID, immunomodulatory drugs; PI, proteasome inhibitors; ACA, alkylating agents; TI, topoisomerase inhibitors; OCA, other cytostatic agents; OI, other immunosuppressive agents.

The public available FEARS database is used to monitor serious adverse events for drugs. A search in the FAERS database was performed by matching cases of MM-associated PML reported from 2002 until 18 May 2022. The search term “progressive multifocal leukoencephalopathy” was used when searching for “reaction type” and yielded 6,165 cases with PML. All cases were listed with suspected product names, active ingredients, and reasons for use of the drug. To identify only MM cases, “myeloma” was used as an additional search term in “reasons for use of drug,” and this search yielded 198 cases with both variables: “Plasma Cell Myeloma” and “PML.” To reduce duplicates, cases were matched based on characteristics. Cases with the same age, gender, and country where the event occurred were matched, if the event date, FDA, or manufacturer report date were within the same 30 days. A detailed listing of matching criteria is found in Supplementary Table. After adjusting for duplicates, the number of cases was reduced to 117, corresponding to a reduction of 40.9%. Relevant-reported MM-specific therapies were included in the analysis. Demographics and therapy characteristics are reported in Table 1. Reported MM-specific therapies in patients with PML from the FAERS database are depicted in Figure 3. The most frequently given therapies were IMiD, alkylating agents (AA), PI, monoclonal antibodies (MAB), topoisomerase inhibitors (TI), other cytostatic agents (OCA), and other immunosuppressive agents, listed based on the frequency of use (shown in Table 1).

**Table 1:** Demographics and therapy characteristics of patients in the United States Food and Drug Administration adverse event reporting system database.

**Population, *n***	**117**
Female	32,4%
Age (years), mean, SD	65.4, 7.5
**Number of drugs pr. patient**	
1	*N* = 33, 28.2%
2	*N* = 32, 27.4%
3	*N* = 24, 20.5%
9-Apr	*N* = 20, 17.1%
>10	*N* = 8, 6.8%
**Proportion of patients treated with following drug classes**	
Immunomodulatory drugs	96.60%
Alkylating agents	52.10%
Proteasome inhibitors	48.70%
Monoclonal antibodies	29.90%
Topoisomerase inhibitors	13.70%
Other cytostatic agents	12.80%
Other immunosuppressive agents	0.90%

## Discussion

This paper describes a case of PML in a patient having MM for more than 16 years. PML is a rare condition in MM, and so far, only 16 cases are described worldwide from 1,965 until now (13). To our knowledge, no studies have described the incidence of PML in patients with MM. This is the first analysis of reported cases of PML in MM from the FAERS database. Through an analysis of the FAERS database, this paper suggets that PML incidence in MM is higher than the 16 described cases, since 117 cases were identified from 2002 until May 2022.

In this case report, PML occurred after a treatment history with nine combined MM therapies. Only one case of PML in a patient with untreated MM is described in the literature, where PML was diagnosed prior to MM diagnosis (14). The FAERS database demonstrated that 72% of patients were treated with two or more different MM specific therapies, before a diagnosis of PML, especially IMiD were given more frequent than any other treatment. This paper suggets that PML in patients with MM is related to treatment with immunosuppressive therapies rather than the MM diagnosis itself and physicians should be aware of this, especially in the late stage of heavily treated MM patients. The analysis of the FEARS database demonstrates that 97% of patients with MM, that developed PML, were treated with IMiD and that AA (52%) and PI (49%) were the second and third most frequent treatments, respectively (Table 1). IMiD is among the most widely used treatments in MM, both in case of maintenance therapy after ASCT and in patients not eligible for ASCT. This could explain that almost all patients that developed PML received IMiD during the disease course. The FAERS analysis showed that PML after treatment with MAB and TI was relatively infrequent compared to IMiD, AA, and PI. This might be explained by the novelty of MAB in MM treatment and that TI is currently less frequently used since other therapies have been prioritized by treatment guidelines (15).

There are limitations when using FAERS as a database for drug-associated PML and for PML incidence in MM. First, healthcare professionals, consumers, and manufacturers submit reports voluntarily to the FDA. This means that there are duplicate reports, and some adverse events are not reported, which is why FAERS cannot be used solely to estimate incidence. Moreover, a report of an adverse event does not establish causation with the reported drug. The event might have been related to the underlying disease or caused by other drugs. The information from the FAERS database solely depends on the reporter. There are incomplete reports in the database that do not contain all the necessary information, and submission of a report does not require medical confirmation. Due to this, FAERS cannot be used as an absolute indicator of drug safety.

There were also limitations in the analysis of the FEARS database. Cases with similar dates that were not matched due to missing information regarding age and gender, could lead to an overestimation of the incidence. Vice versa, cases could have been matched as duplicates wrongly, causing an underestimation of the incidence.

This paper demonstrates that PML in patients with MM is associated with treatment with immunosuppressive therapies, rather than the MM disease itself, and physicians should be aware of this, especially in the late stage of heavily treated MM patients.

## Data availability statement

The datasets presented in this article are not readily available because of ethical and privacy restrictions. Requests to access the datasets should be directed to the corresponding author.

## Ethics statement

Written informed consent was obtained from the spouse of the patient for the publication of any potentially identifiable images or data included in this article.

## Author contributions

EJ and TS conceptualized the manuscript. EJ, RA, PT, and TS contributed to data analysis, interpretation, drafting, and revision of the manuscript. All authors contributed to the article and approved the submitted version.
