# Supplementary material for: Progressive multifocal leukoencephalopathy in a patient with multiple myeloma: a case report and analysis of the FDA adverse event reporting system
Source: Front Neurol. 2023 May 4;14:1098930. doi: 10.3389/fneur.2023.1098930 (PMC10192558; doi:10.3389/fneur.2023.1098930)
Supplement: Supplementary file 1 [file Table_1.DOCX]

# Supplementary

**1.1 Criteria for matching of duplicates in FAERS**

Cases that were assumed duplicates were matched based on the following criteria:

- Identical gender and/or age, country where event occurred and where the event dates were within a range of 30 days
- Identical gender and/or age, country where event occurred and where the initial FDA report OR latest received manufacturer report were dated within a range of 30 days
- Identical gender and/or age, country where event occurred, bodyweight and where the initial FDA report OR latest received manufacturer report were dated within a range of 3 months
- Cases were not matched if the event date was > 3 months apart, regardless if the above-mentioned criteria were met
